# Injuries from border wall falls after 2018 are more severe: a retrospective cohort study

**DOI:** 10.1186/s40621-024-00544-y

**Published:** 2024-11-04

**Authors:** Gregory H. Whitcher, Susan F. McLean

**Affiliations:** 1 Department of Emergency Medicine, Texas Tech University Health Sciences Center El Paso, El Paso, TX USA; 2 Department of Surgery, Texas Tech University Health Sciences Center El Paso, El Paso, TX USA

**Keywords:** Border, Falls, Trauma, Epidemiology, Immigration

## Abstract

**Background:**

The U.S.-Mexico “border wall” between El Paso, Texas and Ciudad Juárez, Mexico was raised and extended beginning in 2018 in accordance with Presidential Executive Order 13,767. We hypothesized that these changes resulted in increased incidence and severity of injuries of individuals attempting to cross the border wall in the El Paso region.

**Methods:**

A retrospective cohort review was conducted of University Medical Center of El Paso Trauma Registry charts from 2001 to 2022. Year of injury, gender, age, Injury Severity Score, hospital length-of-stay, ICU length-of-stay, ventilator days, and survival were analyzed by Chi-square analysis with Fisher’s exact test for categorical variables and Independent Samples T-test for continuous variables. An independent samples Mann Whitney U Test was used to compare border wall fall injuries before and after 2018.

**Results:**

Of the 842 patients reviewed, 69 patients presented before 2018 and 773 presented from 2018 to 2022. Statistically significant differences were identified in the mean Injury Severity Score which increased from 6.3 (SD ± 3.8) to 8.3 (SD ± 5.5, *p* < .001) and the mean hospital length-of-stay which increased from 6.7 days (SD ± 5.5) to 9.5 days (SD ± 8.0, *p* < .005).

**Conclusion:**

The incidence, severity, and hospital length-of-stay related to injuries crossing the U.S.-Mexico border have increased with changes in height of the border wall since 2018. Additional resources should be allocated to Emergency Departments and Trauma Centers along the Southwest Border to serve this unique patient population. Additional consideration should be given to the cost of the border wall.

## Background

A physical barrier between the El Paso, Texas and Ciudad Juárez, México was first created during Operation Hold-the-Line in 1993 (U.S. C.B.P. 2023). Changes in the length and height of the barrier since then can be traced to two key legislative events. First, the Secure Fence Act of 2006 (H.R.6061 2006) and second, President Trump’s Executive Order 13767 of 2017 (The White House 2017).

In November 2005, the Department of Homeland Security (DHS) established the Secure Border Initiative (SBI) to “secure the Nation’s borders and reduce illegal immigration.” Congress amended Sect. 102(b) of the Illegal Immigration Reform and Immigrant Responsibility Act (IIRIRA) with the Secure Fence Act of 2006, requiring reinforced fence construction and accompanying physical barriers in priority areas along the southwest border in California, Texas, New Mexico, and Arizona (Nunez-Neto et al. 2011). The El Paso Sector was one of five regions chosen for the construction of additional border barrier (5 miles west of Columbus, NM to 10 miles east of El Paso, Texas) (H.R.6061 2006). This legislation led to the construction of approximately 90 miles of pedestrian fencing between 2007 and 2011 (United States Government Accountability Office 2017).

**Fig. 1:**
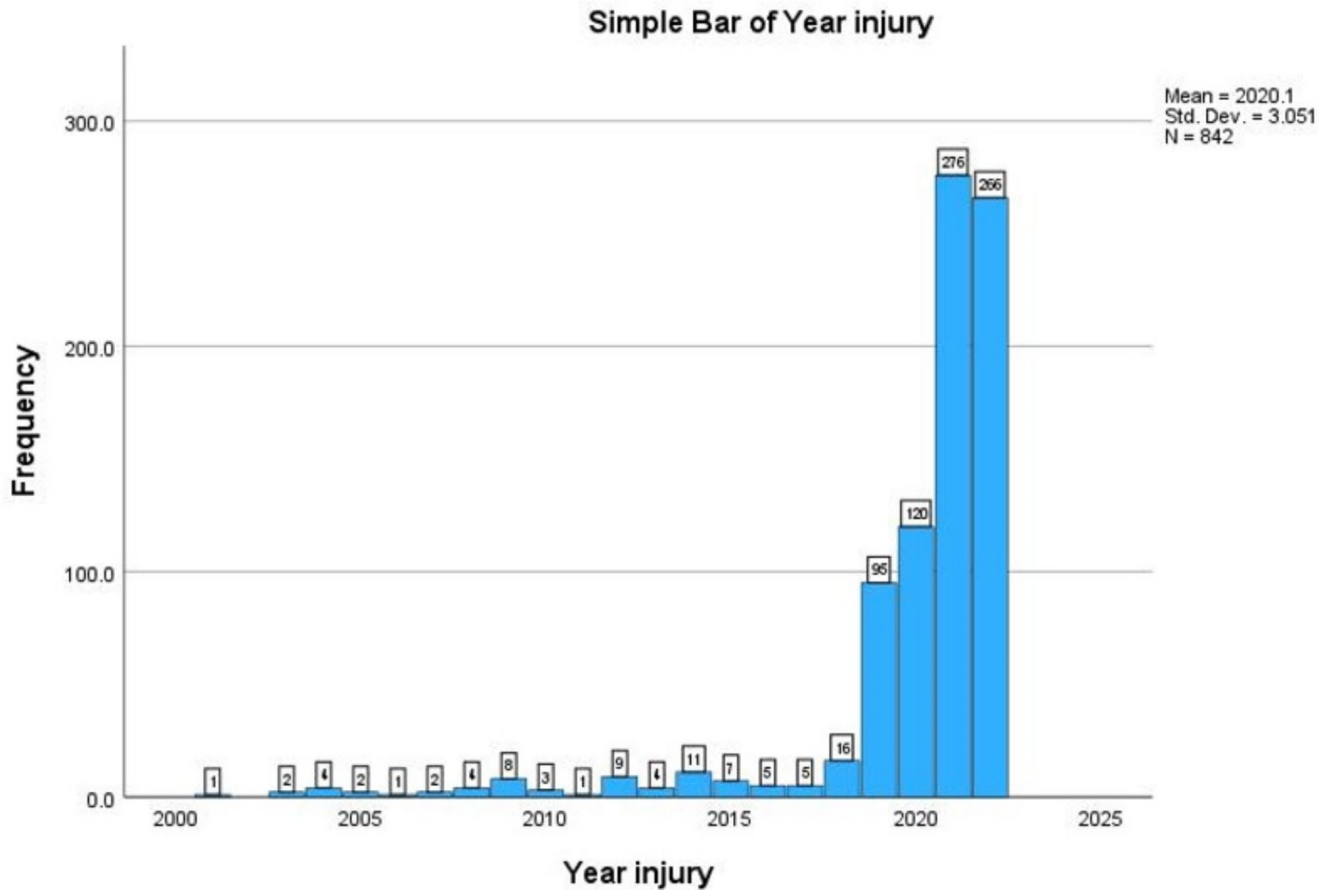
Frequency of border wall fall injuries presenting to UMC-El Paso from 2001 to 2022

By 2016, 82% of the border in Customs and Border Protection’s (CBP) El Paso Sector was fenced and approximately 90 miles (54%) consisted of pedestrian-specific fencing (United States Government Accountability Office 2017). Despite increased security, injuries related to the border fence that were transported to University Medical Center of El Paso remained relatively low at that time (Fig. 1).

**Fig. 2:**
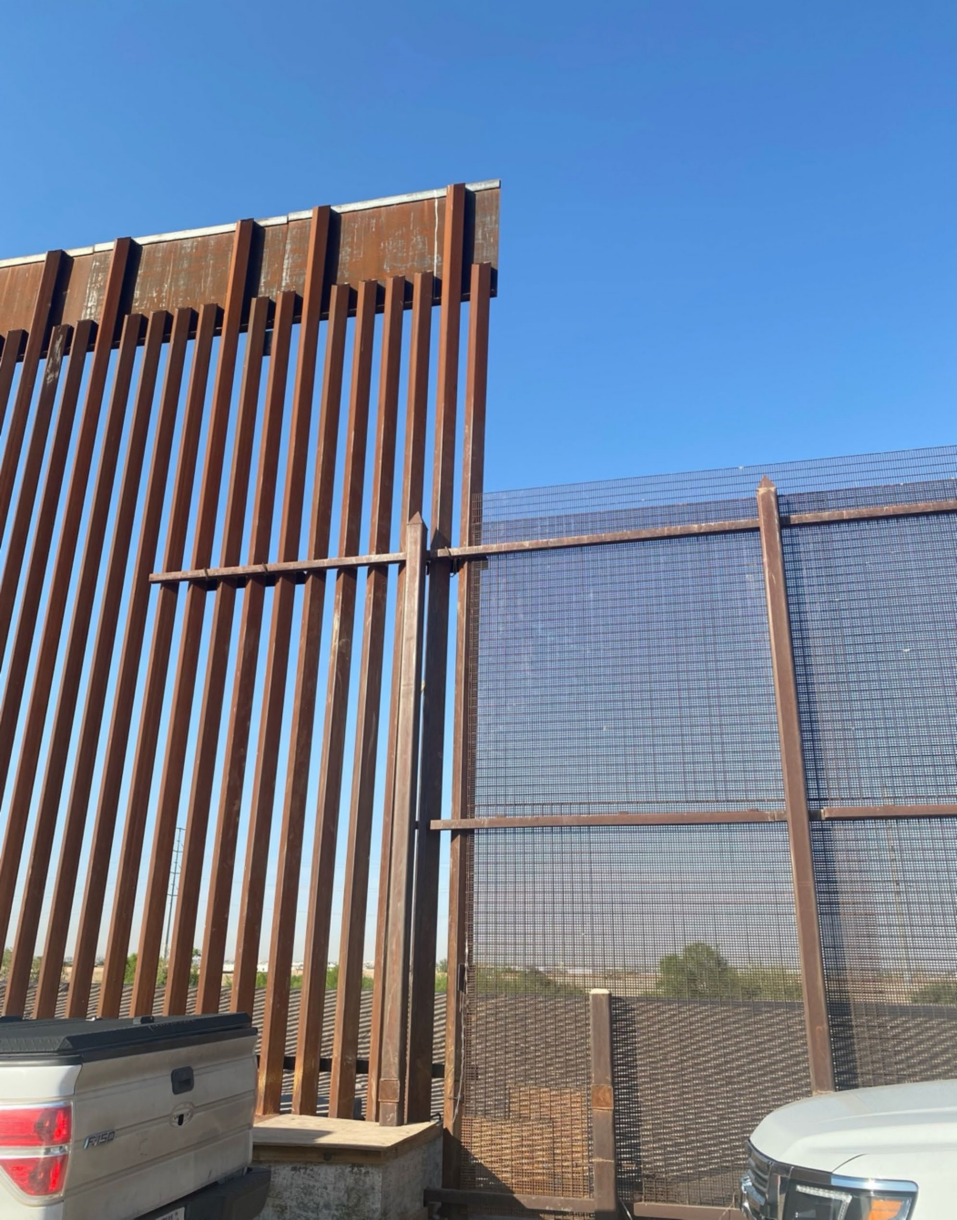
New (left) and old (right) U.S.—Mexico Border Wall, El Paso County, April 2023. Photo by Susan F McLean, MD

In 2017, newly elected President Donald Trump issued Executive Order 13767 which mandated “immediate construction of a physical wall on the southern border” (U.S. C.B.P. 2023). Pursuant to this Executive Order, 20 miles of the border barrier in the El Paso Sector was extended and raised beginning in 2018 (U.S. Department of Homeland Security 2018). Many details of the border wall height are not publicly available but according to documents released by a Freedom of Information Act (FOIA) request, the wall was envisioned to be 30 feet tall and CBP required the wall be greater than 18 feet tall. CBP requested it “not be possible for a human to climb to the top of the wall or access the top of the wall from either side unassisted (e.g. via the use of a ladder, etc.)” (CBP, 2021) (Fig. 2).

Descriptions of US-Mexico border wall falls in the medical literature began in the early 2000s, most notably in 2010 by Kelada et al. (2010). Additional studies (Burk et al. 2017; Ramey et al. 2019; Palacio et al. 2021; Liepert et al. 2022; Polmear et al. 2023) were published between 2017 to and 2023 and continued to be published by groups in California, Arizona and Texas. Based on these publications, a theme emerges that border wall fall victims are young and suffer primarily musculoskeletal or neurological injuries. In many cases, these injuries are disabling. To our knowledge, there have been no studies that compared injury epidemiology before and after the border wall height changed.

Beginning in 2019, the number of patients who presented to University Medical Center of El Paso for border wall related injuries rose dramatically. Changes in wall height and our clinical experience led us to hypothesize that after 2018, there would be increased severity of injuries for individuals attempting to cross the U.S.-Mexico border wall. We also hypothesized that after 2018, there would be a greater frequency of patients presenting to our hospital for border wall fall injuries.

## Methods

After approval from the Texas Tech Health Sciences Center Investigational Review Board for Human Subjects Research (FWA#: 00020736, El Paso IRB #00009945, IRB #: E20096), a data request was sent to the University Medical Center (UMC) Trauma databank for patients with a mechanism-of-injury of “fall” and “border” between January 1, 2000, to December 31, 2022. Patients are added to this registry according to the National Trauma Data Standard (NTDS) Patient Inclusion Criteria (American College of Surgeons, 2022). The UMC Trauma Registry was queried for patient gender, age, ISS, hospital LOS, ICU LOS, ventilator days, survival and COVID-19 test result. A retrospective review was then conducted of the medical records of patients who fell from the border wall near El Paso, Texas and were admitted to the Level 1 Trauma Center.

In this study, Injury Severity Score was used as a marker for overall injury severity. Injury Severity Score (ISS) in the UMC Trauma Registry is calculated based on the “New Injury Severity Score” method described by Osler et al. in 1997 (Osler et al. 1997). The 3 highest AIS scores were squared and summed to yield the ISS reported in this study.

**Fig. 3:**
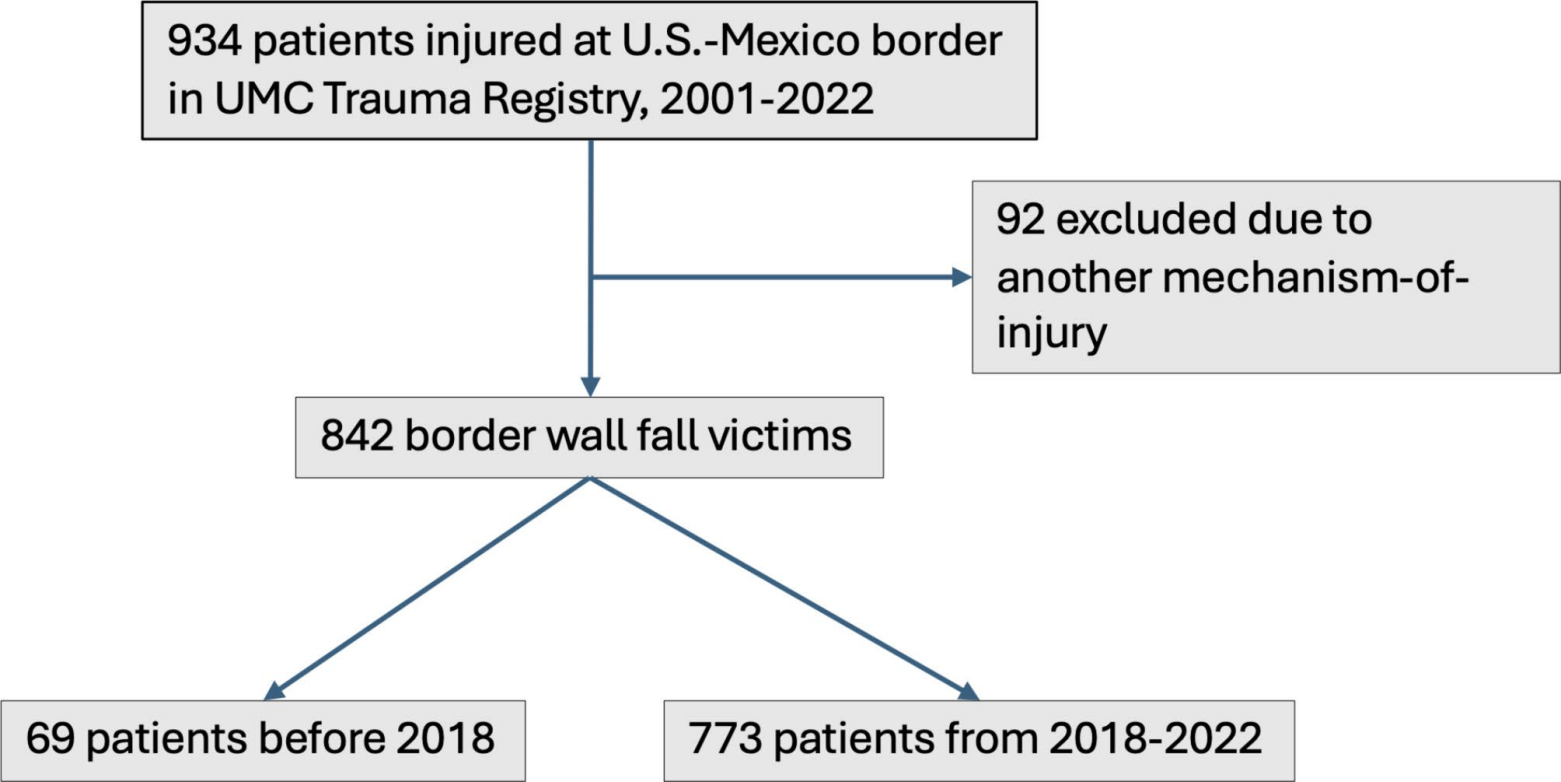
Diagram of border wall fall patient selection

A total of 92 patients were removed from the original dataset due to injuries coded in their medical records that were not exclusively attributable to a fall from the US-Mexico border wall (Fig. 3). These patients had just crossed the border, or were in the process of migrating, and were transported to UMC however their mechanism of injury was not a fall. While they likely did cross the border wall with some exceptions, they did not fall from the border wall and therefore were not counted in the final dataset. Examples of their injuries include cold and heat exposure, snake bites, drownings in the Rio Grande or the canal near the border which is part of the Rio Grande River system. Since the river and canal is immediately next to the border, it is frequent that persons who have just crossed the border are then must cross the canal. Similarly, persons who were pedestrians struck by car or ATV near border were excluded to be analyzed. It is well documented that many persons who have just crossed the border then need to cross the highway next to the border and are struck by passing vehicles.

Categorical variables were analyzed by Chi-square analysis with Fisher’s exact test when appropriate. Group comparison of continuous variables was completed by Independent Samples T-test. When ICU length of stay and ventilator days were calculated, only those patients who went to ICU or were on a ventilator were included. An independent samples Mann Whitney U Test was used to compare border wall fall injuries before and after 2018.

## Results

We identified 842 patients in the UMC Trauma Registry who were admitted to the hospital after a fall from the border wall. Characteristics of the group are shown in Tables 1 and 2. 69 patients fell between 2001 and the end of 2017 and 773 from January 1, 2018-December 31, 2022. Most patients survived (99.4%) but in our cohort there were 5 deaths.

**Table 1:** Demographics of border wall fall patients 2001–2022

Variable	Number	Percent
Males	439	52.1
Females	403	47.9
Total patients	842	

**Table 2:** Demographics and outcomes of all border wall fall patients from 2001–2022

Variable	Number	Mean	Range	Standard deviation
Age (years)	842	32.1	4–72	10.3
ISS	842	8.2	1–42	5.4
Hospital LOS (days)	842	9.24	1–89	7.9
ICU LOS (days)	151	3.91	1–40	5.4
Ventilator days	17	9.5	2–31	0.1
Survival	842	837 survived, 5 died		

**Fig. 4:**
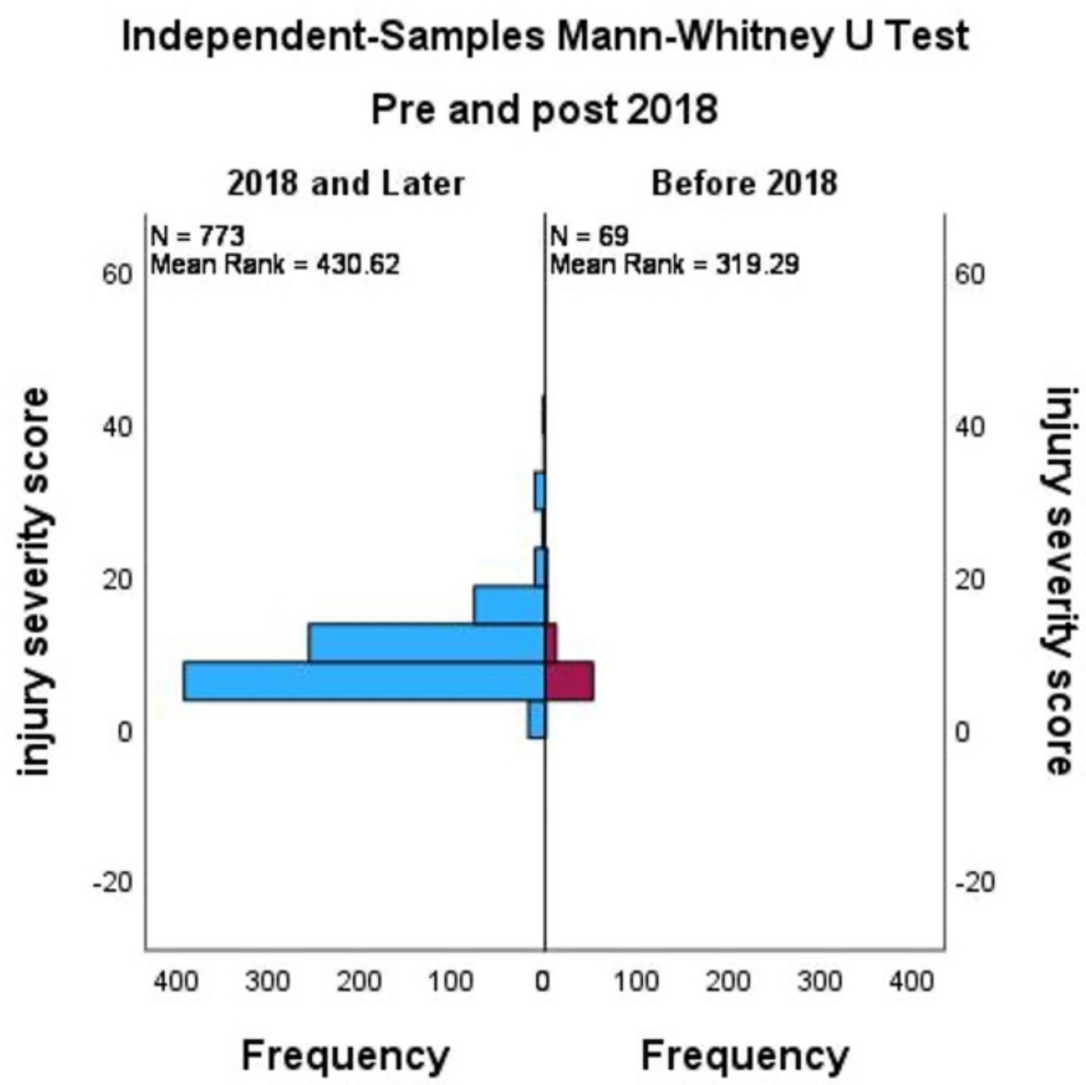
Mann-Whitney U test of injury severity score before and after 2018

The population was split into 2 groups for analysis: before and 2018–2022. The group characteristics as well as the outcomes are presented in Table 3. The two groups had similar demographics including age and gender proportions both prior to and after 2018. The mean ISS increased from 6.3 (SD ± 3.8) before 2018 to 8.3 (SD ± 5.5, *p* < .001) from 2018 to 2022 representing a 32% increase, (*p* < .001) (Tables 3 and 4; Fig. 4). In addition, the mean hospital length of stay (LOS) also increased from 6.7 (SD ± 5.5) days to 9.5 (SD ± 8.0, *p* < .005) days (Table 3). Along with the increased mean hospital days from 2018 to 2022, there was an increase in total hospital days, from 460 total patient-days from 2001 to 2017, and 7316 patient-days after January 1, 2018.

From 2020 to 2022, all admissions were tested for SARS-CoV-2 virus. Prior to 2018 there were no cases of COVID-19 and after 2018 there were 48 patients who tested positive. All 48 patients survived to discharge, with a mean hospital LOS of 14.1 (SD ± 13) days and mean ISS and standard deviation of 9.4 (SD ± 5.7). Both hospital LOS and ISS were higher in the COVID positive patients.

**Table 3:** Demographics and outcomes of patients injured by border wall falls before and after 2018

Variable	Before 2018		After 2018		*p*
	Number	Mean (SD)	Number	Mean (SD)	
Age (years)	69	33.0 (10.5)	773	32.0 (10.3)	0.461
ISS	69	6.3 (3.8)	773	8.32 (5.5)	< 0.001
Males	42	61.0%	397	51.4%	0.134
Females	27	39.1%	376	48.6%	
Hospital LOS (days)	69	6.7 (5.5)	773	9.5 (8.0)	0.005
ICU LOS (days)	12	4.8 (6.0)	139	3.8 (5.3)	0.54
Ventilator days	2	10.5 (12.0)	15	9.3 (9.2)	0.872
Sum hospital days	69	460	773	7316	
Covid test positive	0		48		0.004

**Table 4:** Frequency and proportion of patients injured in border wall falls by year from 2001–2022

Year	Frequency	Percent	Valid percent	Cumulative percent
2001	1	0.1	0.1	0.1
2003	2	0.2	0.2	0.4
2004	4	0.5	0.5	0.8
2005	2	0.2	0.2	1.1
2006	1	0.1	0.1	1.2
2007	2	0.2	0.2	1.4
2008	4	0.5	0.5	1.9
2009	8	1.0	1.0	2.9
2010	3	0.4	0.4	3.2
2011	1	0.1	0.1	3.3
2012	9	1.1	1.1	4.4
2013	4	0.5	0.5	4.9
2014	11	1.3	1.3	6.2
2015	7	0.8	0.8	7.0
2016	5	0.6	0.6	7.6
2017	5	0.6	0.6	8.2
2018	16	1.9	1.9	10.1
2019	95	11.3	11.3	21.4
2020	120	14.3	14.3	35.6
2021	276	32.8	32.8	68.4
2022	266	31.6	31.6	100.0
Total	842	100.0	100.0	

**Table 5:** Group statistics of injury severity score before 2018 and 2018 and later

	*N*	Mean	Standard deviation	Standard error mean
Before 2018	69	6.30	3.770	0.454
2018 and later	773	8.32	5.498	0.198

As shown in Fig. 3, an independent samples Mann-Whitney U Test was used to compare Injury Severity Score of border wall fall injuries before and after 2018 and reached statistical significance (*p* < .001). Group statistics of ISS before and after 2018 are shown in table format in Table 5.

Table 6 lists numbers of patients, number of deaths, mean ISS ± SD and hospital length of stay of patients excluded due to a mechanism of injury other than border wall fall. There were 8 total deaths in the dataset of patients who were excluded to be analyzed. In 2020, there were no deaths in the patients excluded. In 2021, two that were excluded died after an MVC and 1 was after accidental drowning and 1 was a passenger injured by boarding a pickup truck or van in a non-collision transport accident after crossing border. In 2022, 4 patients died after migration and were excluded. The mechanism of injury for these deaths was 1 patient died from accidental drowning, and 3 were injured as pedestrians struck by motor vehicle. Mean ISS of all of those removed ranged from 10.5 to 14.8. Mean length of stay for these patients who were removed was 5.5 (SD ± 13.7).

**Table 6:** Analysis of numbers, deaths, survival, ISS and hospital LOS data for patients excluded from analysis

Year	Patients, number	Died, number	Survived, number	ISS (SD)	ISS range	LOS hospital (SD)
2022	37	4	33	10.5 (± 8.1)	1–30	7.8 (± 9.5)
2021	44	4	40	14.8 (± 9.5)	1–38	13.7 (± 15.4)
2020	11	0	11	10.8 (± 3.9)	5–17	5.5 (± 6.6)
Total	92	8	84	5.5 (± 13.7)	1–38	

##  Discussion

Our study demonstrates that since changes in the height of the US-Mexico border wall in the El Paso region in 2018, there were significantly more patients injured (69 vs. 773) with more severe injuries (ISS 6.3 (SD ± 3.8) vs. 8.3 (SD ± 5.5, *p* < .001) and they were hospitalized for a greater length of time (Median hospital LOS 6.7 (SD ± 5.5) days to 9.5 (SD ± 8.0) days (*p* < .005). This study builds on the work of other research groups who have described the injury epidemiology of the border region.

The epidemiology of the border crossing population was first described in 2010 by Kelada et al. (2010). These authors showed that the incidence of injuries while crossing the U.S.-Mexico border at San Diego increased between 2000 and 2007. The mean Injury Severity Score (ISS) of patients in their study was 8.45 (SD ± 5) (Palacio et al. 2021).

Next, Burk et al. showed that for a trauma center at the Arizona border, injuries that occur because of the border barrier are similar to those that occur from other falls from height (Burk et al. 2017). The authors reviewed cases of 174 patients who presented between 2004 and 2010 after falls from the U.S.-Mexico border fence. An association between calcaneal fractures and pilon fractures with thoracolumbar fractures was demonstrated (Liepert et al. 2022).

In 2019, Ramey et al. published a retrospective review of 64 patients identified as falling or jumping from the U.S.-Mexico border wall who also sustained cranial and or spinal trauma based on Banner University of Arizona Medical Center Tucson records from 2012 to 2017 (Ramey et al. 2019). As seen in all other studies of this population, including our own, injuries from the U.S.-Mexico border wall predominantly affect young people with average age reported to be 35 years (range 14–63). This was also observed in our cohort where the mean age of injured patients was 32 years (range 4–72, Table 2).

Palacio et al. conducted a retrospective study of 178 undocumented immigrants injured while crossing the U.S.-Mexico border from the trauma registry of a Level II trauma center in the Rio Grande Valley between 2014 and 2019 (Palacio et al. 2021). Like the trends seen in other studies, border wall fall victims in their study primarily suffered from extremity injuries (60.7%) followed in frequency by neurological injuries.

Liepert et al.. published a research letter showing a dramatic rise in the number of trauma center admissions in San Diego, California attributable to injuries sustained from falls off the U.S.-Mexico border wall beginning in 2019 (Liepert et al. 2022). The data from our study agree with their assertion that “raising the U.S. border wall to 30 feet is associated with increased deaths, increased ISS, and increased health care costs.”

In 2023, Polmear, et al. published a research study of 448 patients injured by border wall falls that were admitted to a trauma center on the U.S.-Mexico border between 2016 and 2021 (Polmear et al. 2023). Some of the patients included in that study are likely included in the present study. 414 patients (92.4%) in their cohort required a surgical procedure. The authors point out that the median monthly frequency of admissions due to border wall falls increased in their cohort in 2021. Median ISS in their study was 9 (IQR 7, range 1 to 75). The authors also point out that this patient population faces significant obstacles to optimal follow-up for injuries sustained but provided thoughtful recommendations to address the unique obstacles faced by this patient population.

**Fig. 5:**
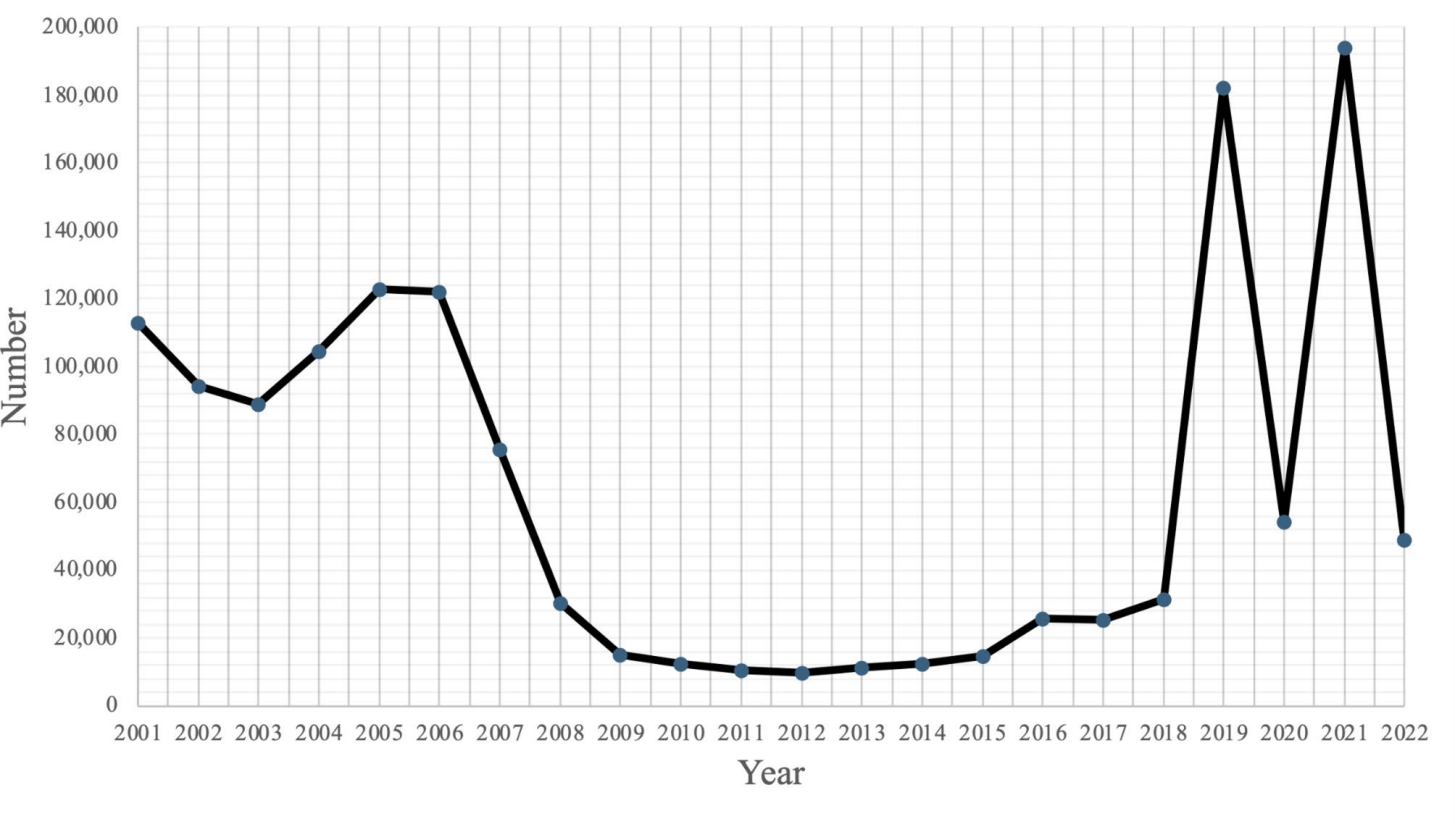
US B.P. Encounters El Paso Sector: 2001–2022

Both Liepert et al. and Polmear, et al. use CBP apprehensions as a surrogate for border crossings. In the latter study, a linear regression between US CBP apprehensions and hospital admissions was statistically significant. However, apprehensions may be a poor surrogate for the true number of attempts at immigration due to both repeated attempts and incomplete sampling of the population of interest. Importantly, fluctuations in US B.P. encounters outside ports-of-entry have been reported (Department of Homeland Security 2022) during other periods in recent history (Fig. 5) but were not accompanied by similar increased numbers of trauma patients presenting to our trauma center (Fig. 1). Given the surreptitious nature of border crossing, we believe no good surrogate currently exists to normalize the increase of injuries. Uncertainty around the total number of individuals crossing the U.S.-Mexico border will likely continue to pose a challenge for groups studying this patient population. Publicly available information on how surveillance methods may affect discovery of border wall fall injuries is limited. This is due to the exploitable nature of information on surveillance methods and thus its protection from FOIA requests.

To our knowledge, there were no changes in Border Patrol personnel or the local trauma system (i.e. EMS) that can account for the increased number of patients injured by border wall falls. In the case of border wall falls, first responders are typically US B.P. agents. In publicly available DHS data, the number of U.S. Border Patrol agents on the southwest border has not significantly changed since the period from 2005 to 2009. El Paso Sector-specific B.P. agent staffing information is not available.

The efficacy of the border wall towards its intended purpose (deterring illegal immigration) is not within the scope of this study. As others have described (Kelada et al. 2010; Burk et al. 2017; Ramey et al. 2019; Palacio et al. 2021; Liepert et al. 2022; Polmear et al. 2023), we show that height increases in the U.S.-Mexico border wall that occurred since 2018 have resulted in increased incidence of injuries, severity of injuries and length of hospitalization from border wall injuries.

Our study is flawed by several factors. First, survivor bias is imparted by surveying hospital patients without including individuals who either died in the field or were not immobilized and were thus not transported to our Trauma Center. Second, patients discharged from the ED may meet not NTDS criteria and thus are not always included in these data. This may systematically skew our results suggesting a more severe pattern of injuries from border wall falls overall.

The height of a victim’s fall reported in the data provided by the UMC Trauma Registry was based on the victim, CBP agent or EMS personnel’s report included in hospital documentation. Importantly, bystander approximations of height have been shown to be inaccurate. In 2018, Carey et al. showed that for heights greater than 15 ft, less than 50% of study participants could correctly identify the height of a horizontal line (Carey et al. 2018). Therefore, we avoided drawing conclusions on the height of fall for the victims in this study based on the medical record.

Because our data was obtained from a heterogenous patient registry, our analysis could have been affected by sampling error. A total of 92 patients were manually removed from the dataset during analysis when we found their injuries were not exclusively attributable to a fall from the US-Mexico border wall. The excluded population included individuals with cold and heat injuries, snake bites, gunshot wounds, drownings and those who were struck by cars or an ATV (Table 6). These types of injuries are not unusual for migrants as the border is immediately adjacent to the Rio Grande River which in some areas passes through a canal and in other areas is next to roadways.

Last, our study relies on the use of ISS which is an imperfect scoring system. ISS does not account for multiple injuries to the same body region. In the ISS, many injuries carry the same weight, which does not consider disproportionate effects that certain body system injuries have on patients (e.g., severe Traumatic Brain Injury does not necessarily impart the same effect as a severe musculoskeletal injury). In other words, while musculoskeletal injuries are often not life-threatening, they are life-altering and confer significant morbidity and therefore we assert, are significant to patients.

## Conclusion

The incidence and severity of injuries related to crossing the U.S.-Mexico border have increased since 2018 with changes in height of the border wall. Along with the increased injury severity, hospital length of stay increased. Additional resources should be allocated to Emergency Departments and Trauma Centers along the Southwest Border to serve this unique patient population. Additional consideration should be given to the cost of the border wall.

## Data Availability

The datasets used and/or analyzed during the current study are available from the corresponding author on reasonable request.

## Abbreviations

ATV: All-Terrain Vehicle
BWF: Border wall fall
CBP: Customs and Border Protection
DHS: Department of Homeland Security
FOIA: National Freedom of Information Act
ICU: Intensive Care Unit
IIRIRA: Illegal Immigration Reform and Immigrant Responsibility Act
IRB: Investigational Review Board for Human Subjects Research
ISS: Injury Severity Score
LOS: Length of Stay
MOI: Mechanism-of-injury
NTDS: National Trauma Data Standard
SBI: Secure Border Initiative
SD: Standard Deviation
UMC: University Medical Center
